# Comparison of cognitive functional therapy and movement system impairment treatment in chronic low back pain patients: a randomized controlled trial

**DOI:** 10.1186/s12891-023-06815-x

**Published:** 2023-08-29

**Authors:** Sahar Nazary Soltan Ahmad, Amir Letafatkar, Britton W. Brewer, Ali Sharifnezhad

**Affiliations:** 1 Faculty of Physical Education and Sport Sciences, Department of Biomechanics and Sport Injuries, Kharazmi University, Tehran, Iran; 2 Department of Psychology, Springfield College, Springfield, USA; 3 Department of Sport Biomechanics and Technology, Sport Science Research Institute, Tehran, Iran

**Keywords:** Chronic low back pain, Cognition, Exercise therapy, Gait kinetics, Kinesiophobia

## Abstract

**Background:**

This study aimed to compare the effects of cognitive functional therapy (CFT) and movement system impairment (MSI)-based treatment on pain intensity, disability, Kinesiophobia, and gait kinetics in patients with chronic non-specific low back pain (CNSLBP).

**Methods:**

In a single-blind randomized clinical trial, we randomly assigned 91 patients with CNSLBP into CFT (n = 45) and MSI-based treatment (n = 46) groups. An 8-week training intervention was given to both groups. The researchers measured the primary outcome, which was pain intensity (Numeric rating scale), and the secondary outcomes, including disability (Oswestry disability index), Kinesiophobia (Tampa Kinesiophobia Scale), and vertical ground reaction force (VGRF) parameters at self-selected and faster speed (Force distributor treadmill). We evaluated patients at baseline, at the end of the 8-week intervention (post-treatment), and six months after the first treatment. We used mixed-model ANOVA to evaluate the effects of the interaction between time (baseline vs. post-treatment vs. six-month follow-up) and group (CFT vs. MSI-based treatment) on each measure.

**Results:**

CFT showed superiority over MSI-based treatment in reducing pain intensity (P < 0.001, Effect size (ES) = 2.41), ODI (P < 0.001, ES = 2.15), and Kinesiophobia (P < 0.001, ES = 2.47) at eight weeks. The CFT also produced greater improvement in VGRF parameters, at both self-selected (FPF[P < 0.001, ES = 3], SPF[P < 0.001, ES = 0.5], MSF[P < 0.001, ES = 0.67], WAR[P < 0.001, ES = 1.53], POR[P < 0.001, ES = 0.8]), and faster speed, FPF(P < 0.001, ES = 1.33, MSF(P < 0.001, ES = 0.57), WAR(P < 0.001, ES = 0.67), POR(P < 0.001, ES = 2.91)] than the MSI, except SPF(P < 0.001, ES = 0.0) at eight weeks.

**Conclusion:**

This study suggests that the CFT is associated with better results in clinical and cognitive characteristics than the MSI-based treatment for CNSLBP, and the researchers maintained the treatment effects at six-month follow-up. Also, This study achieved better improvements in gait kinetics in CFT. CTF seems to be an appropriate and applicable treatment in clinical setting.

**Trial registration:**

The researchers retrospectively registered the trial 10/11/2022, at https://www.umin.ac.jp/ with identifier number (UMIN000047455).

**Supplementary Information:**

The online version contains supplementary material available at 10.1186/s12891-023-06815-x.

## Background

Low back pain (LBP) affects people around the world at a high occurrence rate [1]. Chronic Low back pain causes a significant amount of medical expenses, work absenteeism, and disability and associates with multidimensional factors such as biopsychosocial factors and musculoskeletal impairments [2].

One important impairment in patients with LBP is related to the walking task. Pain, kinesiophobia, and effort to reduce pain by restricting spinal movement cause changes in gait parameters such as decreased step length and step speed, along with widened step width in patients with chronic LBP [3]. The influence of LBP on vertical ground-reaction force (VGRF) is not however clear. In a gait analysis study, pain-free individuals and patients with LBP showed no difference in VGRF parameters; while, patients with LBP and lower limb pain showed significant decreases of all parameters (apart from the first peak force) when walking at their preferred speed [4]. In a randomized controlled trial (RCT), researchers investigate that LBP patients use strategies to reduce the amount of force imposed on their body and the Pilates intervention can improve weight discharge during walking and reduce pain compared with no intervention [5].

Current management of chronic LBP includes a range of different treatments [6–9]. Over the past decades, researchers have advocated a variety of non-drug therapies, including motor control exercises, movement re-education, psychosocial-based intervention, cognitive functional therapy (CFT) and/or movement system impairment (MSI)-based treatment [10, 11]. However, despite applying various treatment approaches, conservative treatment for chronic non-specific LBP (CNSLBP) yields small effect sizes. The reasons behind failure of current clinical practice might lie in not considering multidimensional biopsychosocial factors and person-centered supervised exercise program [12, 13].

A movement-based strategy, uniquely designed for each patient, suggests that the MSI-based treatment approach improves the accuracy of spinal movements and induces specific changes in the musculoskeletal system, such as altering movement patterns and promoting painless movement patterns [14, 15]. In this approach, repetitive movements and sustained postures can affect characteristics of the tissues in ways that make the body more susceptible to healing processes [16]. MSI-based treatment approach aims to improve the patient’s ability to control the trunk and stabilize the spine during activities of daily living, isolated trunk movements, or trunk movements induced by limb movement, thereby decreasing aberrant trunk movement patterns during voluntary movements. However, this approach mainly lacks consideration of the psychological and behavioral factors related to CNSLBP, focusing mainly on movement patterns(e.g., fear avoidance behavior).

On the other hand, CFT is a novel approach that not only cover postural and movement behaviors, but also consists of two other components includes biopsychosocial understanding of pain, and lifestyle change [2]. To address the multidimensional nature of CNSLBP, this training utilizes information from a clinical reasoning framework [2]. One study among people with CNSLBP reported CFT approach was more effective than combining manual therapy and core exercise in disability after eight weeks but the treatment effect didn’t last in 6-month and one year follow ups [17] on the other hand Vibe Fersum et al. reported CFT was more effective than manual therapy and exercise at reducing disability and pain-related fear, at 3-year follow-up in people with non-specific chronic low back pain [18]. In a multiple case-cohort study, researchers showed that CFT significantly improved pain, disability, and psychological outcomes. However, the authors recommended comparing CFT with other conservative interventions for the management of CNSLBP in larger RCTs [19].

According to the above-mentioned statements, this RCT aimed to compare the MSI-based treatment with the CFT approach on pain intensity, disability, Kinesiophobia, and gait kinetics in patients with CNSLBP. We hypothesized that CFT could improve pain, disability, and Kinesiophobia, while MSI-based treatment seemed more influential for gait kinetics.

## Method

### Design overview

This was a 2-arm RCT, parallel-group study with blind outcomes assessor, conducted from September 2018 to August 2019 in the corrective exercises center of the Kharazmi University, Tehran, Iran. The trial was retrospective registered 10/11/2022, at https://www.umin.ac.jp/ with identifier number (UMIN000047455).

The researchers measured pain intensity as the primary outcome using the Numeric Ratings Scale (NRS) and assessed changes in disability, Kinesiophobia, and VGRF parameters as secondary outcomes with the Oswestry Disability Index (ODI), Tampa Kinesiophobia Scale (TKI), and (Force distributor treadmill), respectively, a week before the interventions. After assessing demographic data and outcome measures, the researchers randomly assigned patients to receive either CFT or MSI-based treatment.

### Setting and study population

The biomechanics laboratory of the Sport Sciences Research Institute conducted baseline, post-treatment, and follow-up assessments. A well-experienced assessor assessed the outcomes at the baseline, the end of the 8-week training period (post-treatment), and the end of the six-month follow-up in the same place. The patients did not receive any treatment, including physiotherapy and exercise, between the post-treatment and the six-month follow-up. The supervised clinical sessions in the corrective exercises center of the university also performed the treatments.

The patients complained LBP were recruited through flyers in the physical therapy clinics, occupational health services, and primary care practices. Based on the inclusion criteria, the researchers enrolled eligible patients to participate in the study.

This study included patients with LBP((according to the criteria of the International Classification of Diseases, 10th Revision [ICD-10] (online website https://icd.who.int/)), classified by the duration of pain for more than 12 weeks (Non-specific LBP) [20] and over 18 years of age, who were eligible to participate.

If the researchers found neurological disorders, spinal pathologies, structural deformity, osteoporosis, inflammatory disorder (e.g., spondylitis), radicular syndrome, history of tumor and fracture in the spine, true leg length discrepancy more than 20 mm, or BMI greater than 30 kg/m2, they excluded the patients.

Based on previous studies, researchers calculated that they should enroll at least 40 subjects per each group to complete the study if they used an α error of 0.05, standard deviation of 2.5, and a power of 0.8 [21, 22]. The G*Power software performed the sample size calculation, considering a mixed-model ANOVA test to analyze the data. The sample size calculation utilized the effect size to estimate the magnitude of the difference between the treatment groups.

### Randomization

A researcher with no involvement in the trial conducted randomization based on a computer-generated random sequencing (in a 1:1 allocation ratio) using the website http://randomizer.org. The researcher arranged the allocation sequence using a blocked randomization model with a block size of 4 and concealed it in numbered, sealed, opaque envelopes. Just before the first treatment session, the treating clinicians opened the envelope to reveal the group allocation.

### Outcome measures

#### Pain intensity

The researchers measured pain intensity using a numerical rating scale (0–10), where 0 signified no pain, and 10 represented the worst unbearable pain. The patients rated their current level of pain intensity using this scale. The numeric rating scale version used to measure pain intensity has not been cross-culturally adapted for the Persian-speaking population with low back pain. Although the NRS is a widely accepted and validated tool for evaluating self-reported pain intensity in various populations [23]. Researchers consider 2 points on the NRS as a minimum clinically important difference (MCID) for patients with chronic low back pain [24].

#### Disability

This study used the Persian translated Oswestry Disability Index to assess disability in patients with low back pain. It is important to note that the researchers cross-culturally adapted the Oswestry Disability Index version for Persian-speaking patients with LBP in this study [25]. This adaptation ensures that this particular cultural and linguistic context uses the questionnaire relevant and appropriate. The questionnaire, which assesses physical disability for LBP and includes pain intensity, patient personal care, lifting, walking, sitting, standing, sleeping, social life, and traveling, utilizes a valid and reliable approach (ICC = 0.80). Patients rated each section, giving a score of 0 to the first statement and 5 to the last statement. Higher scores indicate more physical disability [25, 26]. The range of 2.5 to 5 points encompasses the MCID of Oswestry Disability Index [27]. However, based on the patients’ level of disability, those with minimal disability may clinically deem a change of 1–2 points in scores important, whereas patients with high levels of disability may necessitate a change of 7–8 points [27].

#### Kinesiophobia

One study used the Persian translation of Tampa Scale for Kinesiophobia to evaluate kinesiophobia. This specific version has been cross-culturally adapted for the Iranian patients with chronic persistent pain [28]. This contains 17 items related to pain, fear of movement and re-injury The score ranges from 17 to 68, and the higher scores indicate greater pain, fear of movement, and re-injury. Researchers have demonstrated the reliability and validity of this scale (ICC = 0.77 to 0.78) [29]. The MCID for individuals with chronic musculoskeletal pain are 4.5 points [30].

#### Vertical ground reaction force parameters

Based on the study of da Fonseca et al. (2009) we assessed VGRF parameters [5]. After identifying patients’ dominant leg, we assessed VGRF parameters. Patients performed barefoot walking for at least 10 min at self-selected and faster speeds on the force distributor treadmill (SCHEINWORKS FDM 1.0 model). We recorded VGRF parameters for the dominant leg at baseline, post-treatment, and follow-up, normalizing them to body mass percent. The sampling frequency of treadmill data was standardized at 1000 Hz. Initially, patients’ self-selected walking speed and whether or not patients could walk in a faster speed (5.5 km/h or faster) were determined. Patients started walking on the treadmill at an initial speed of 1 km/h, and every 30 s the speed was increased 0.5 km/h. The researchers instructed them to report the speed at which they walked most conveniently (determined as the self-selected walking speed). In the next stage, patients walked on the treadmill up to the fastest speed of 5.5 km/h or as fast as they could. Then, the treadmill speed was decreased by 1 km/h every 30 s to the final speed of 1 km/h, and then stopped. Patients were asked to rest 5 to 10 min and repeat self-selected walking again, as the previous instruction. After 10 min of walking, when the adaptation was given, the first data collection was collected at the self-selected walking speed. Right after the first collection, the treadmill speed was increased until the fastest speed of 5.5 km/h or up to the maximum speed the participant was able to achieve. Then, data were collected at the fastest speed [5]. The VGRF parameters were defined as follows:

First (FPF) and second Peak Force (SPF): the first and second maximum peak of VGRF during the stance phase in straight walking after the heel contact. Mid-Support Force (MSF): the minimum peak of VGRF between the first and second peak forces. Weight Acceptance Rate (WAR): the magnitude of the first peak force divided by the time at which it occurred. Push-Off Rate (POR): the magnitude of the second peak force divided by the time from the second peak force until the end of the stance phase [5].

### Applied interventions

#### Movement system impairment-based treatment

The movement system impairment-based treatment group received 11 sessions of MSI-based treatment over the 8 weeks for 60 min per session with a supervision of a native speaker experienced (above 5 years) physical therapist with the knowledge of MSI-based treatment [31]. The researchers designed the MSI-based treatment uniquely for each patient based on the interview, clinical examination, and questionnaires, just like they did with the CFT intervention. First, they administered standardized tests to characterize changes in the patient’s low back pain symptoms, and then they modified the treatment to make it more specific based on the participant’s individual symptoms. Depending on the participant’s direction-specific low back pain classification, they performed the intervention following one of the five MSI subgroups namely [1] rotation, [2] extension, [3] flexion, [4] rotation with extension, and [5] rotation with flexion. Finally, Patients treated using the standardized MSI protocol as follows: [1] education regarding normal postures and movements such as sitting, walking, bending, standing, and lying down; [2] education regarding exercises to perform trunk movements as painlessly as possible; and [3] prescription of functional exercises to improve trunk movement [32].

#### Cognitive functional therapy

Cognitive functional therapy was prescribed for each patient in CFT group based the CFT protocol conducted by O’Sullivan et al. (2015) [19, 33]. Patients received supervised 12 sessions of training over the 8-week period with 60 min per session provided with another physical therapist who had been trained in CFT treatment. In this protocol, a physical therapist with more than 5 years of experience conducted an interview and physical examination of the patients to determine their own unique training programs, considering modifiable cognitive, biopsychosocial, functional, and lifestyle behavior factors. The intervention consists of the following 3 main stages: [1] making sense of pain that is completely reflective, where physical therapist could use the context of the patient’s own story to provide a new understanding of their condition and question their old beliefs [2] exposure with control which is designed to normalize maladaptive or provocative movement and posture related to activities of daily living that is integrated into each patient’s functional impairments, including teaching how to relax trunk muscles, how to have normal body posture while sitting, lying, bending, lifting, moving, and standing, and how to avoid pain behaviors, which aims to break poor postural habits; and [3] lifestyle change which is investigating the influence of unhealthy lifestyles in the patient’s pain context. Assessing the individual’s body mass, nutrition, quality of sleep, levels of physical activity or sedentary lifestyle, smoking, and other factors via video calls. Identifying such lifestyle factors helped us to individually advise and design exercise programs, rebuild self-confidence and self-efficacy, promote changes in lifestyle, and design coping strategies.

### Statistical analysis

A statistician performed statistical analyses, using SPSS version 16.0 (SPSS Inc., Chicago, IL, USA). The Kolmogorov-Smirnov and Levene tests were used to check the normal distribution of variables and homogeneity of variance, respectively. We used mixed-model ANOVA to evaluate the effects of the interaction between time (baseline vs. post-treatment vs. 6-month follow-up) and group (CFT vs. MSI) on each measure. We used Bonferroni correction to assess changes in each group across the three-time points. For each variable, we calculated the percentage of change compared with baseline. We also conducted intention-to-treat analyses for all randomized patients, including those who dropped out. Cohen’s d was used to calculate effect sizes (ES), which were classified as small (d < 0.20), medium (d = 0.21–0.79), or large (d > 0.80) [34].

## Results

**Fig. 1:**
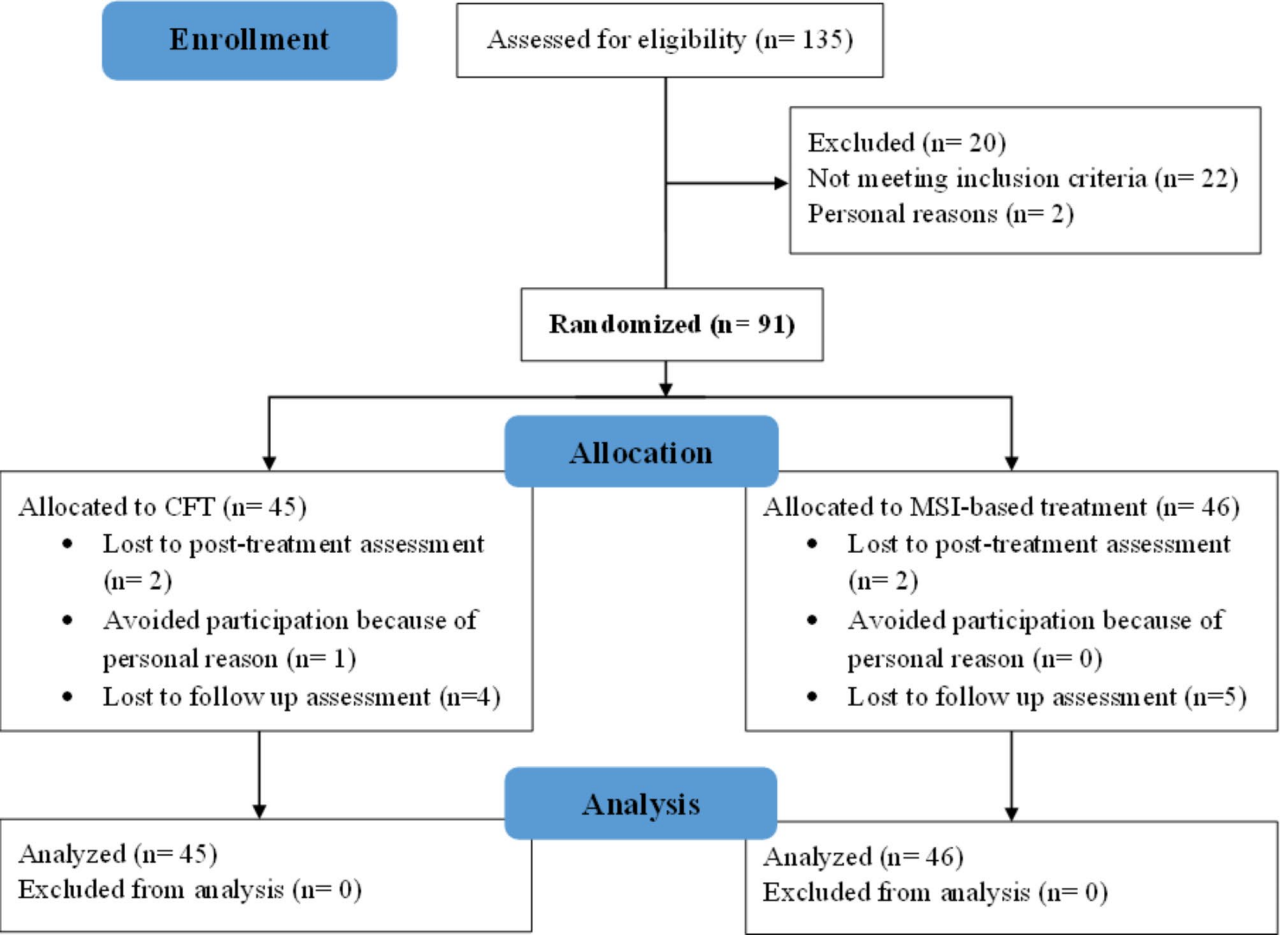
CONSORT Diagram. Flow diagram graphically describes the design of the study: enrolment, intervention, follow-up and data analysis

The CONSORT 2010 guidelines display the flow diagram of this trial in Fig. 1. Initially, researchers assessed a total of 135 patients for eligibility, and then they randomly allocated 91 eligible patients into either the CFT group or the MSI group. Finally, 77 patients completed the study. (38 patients in the CFT group and 39 patients in the MSI-based treatment group).

Table 1 shows the clinical and sociodemographic features of the CFT group and the MSI-based treatment group. Based on the multiple comparison adjustment summarized in Table 2, there was a significant group × time interaction for NRS (*F* = *117.29, p < 0.001), ODI (*F *= 85.84*, *p* < 0.001) and TSK (*F* = *64.93*, *p* < 0.001). Between groups analysis showed significant differences at 8-weeks post-intervention (NRS [*p* < 0.001], ODI [*p* < 0.001] and TSK [*p* < 0.001]) also the values were significantly lower in NRS, ODI and TSK in the CFT group(Mean Difference [MD]= -1.99; [95% CI, (-2.34 to -1.65] for NRS, MD= -9.67; [95% CI, -11.46 to -7.88] for ODI and MD= -9.27; [95% CI, -10.83 to -7.70] for TSK). At 6-months follow-up there was a statistically significant difference (NRS [*p* < 0.001], ODI [*p* < 0.001] and TSK [*p* < 0.001]), where the values were significantly lower in NRS, ODI and TSK in the CFT group (MD= -2.03; [95% CI, (-2.35 to -1.72] for NRS, MD= -14.08; [95% CI, -15.05 to -13.11] for ODI and MD= -11.39; [95% CI, -12.81 to -9.98] for TSK). These results suggest that the CFT protocol was more effective than MSI in reducing pain intensity, disability, and kinesiophobia. The mean of changes in for NRS, ODI, and TSK in both group were more than MCIDs, except for ODI in the follow up. However, in the group of CFT, thses changes were higher as follow: NRS (CFT: 4.17 from basline to post-intervention and 2.66 from basline to follow up, MSI: 2 from basline to post-intervention and 0.91 from basline to follow up), ODI (CFT: 21.09 from basline to post-intervention and 19.96 from basline to follow up, MSI: 10.66 from basline to post-intervention and 5.12 from basline to follow up), and TSK (CFT: 19.35 from basline to post-intervention and 15.95 from basline to follow up, MSI: 11.19 from basline to post-intervention and 5.06 from basline to follow up).

**Table 1:** Baseline demographic data

Clinical and sociodemographic features	CFT (N = 45)	MSI (N = 46)
Gender		
Male Female	17 28	22 26
Leg dominance (n)		
Right left	39 6	38 8
Mean age (SD), years	26.00 (3.17)	27.17(5.10)
Mean stature (SD), cm	164.11(4.22)	163.36(3.28)
Mean body mass (SD), kg	67.40(9.30)	65.04(7.50)
Mean BMI (kg/m2)	25.00(3.09)	24.37(2.75)
Mean duration of LBP (SD), months	8.80 (2.07)	8.43 (1.64)
Running speed		
Self-selected (km/h) Faster (km/h)	3.32(0.28) 5.24(0.26)	3.36(0.29) 5.15(0.25)
Mean true leg length (SD), cm		
Right Left	86.64(2.45) 86.08(2.70)	86.19 (2.00) 85.69(2.10)
Pain medication use in last week, n (%)	8 (17.8)	9 (19. 6)
Active exercise ≥ 2 days in last week, n (%)	18 (40)	20 (43.5)
Smoking, n (%)	3 (6.7)	4 (8.7)
Educational level, n (%)		
Illiterate School level Above bachelor	0 16 (35.6) 29 (64.4)	0 14 (30.4) 32 (69.6)
Effect of LBP on daily activity, n (%)		
Decreased activity ≥ seven days in past four weeks	6 (13.3)	5 (10.9)
Missed work or school ≥ 1 day in past four weeks	2 (4.4)	1 (2.2)

CFT = Cognitive Functional Therapy, MSI = Movement System Impairment, BMI: Body Mass Index, LBP: Low Back Pain. Data are presented as means (± SD) or absolute frequency (percent).

**Table 2:** Changes related to the scores of pain, disability, and kinesiophobia

Characteristic	CFT		MSI		Group Difference, Mean (95% CI)	Effect Size^†^	Main Effect of Time		Interaction Effect		Bonferroni post-hoc tests		
	Means (± SD)	Change relative to baseline (%)	Means (± SD)	Change relative to baseline (%)			*F*	*P-*value	*F*	*P-*value	Time	Group	
NRS (0–10)													
Baseline	6.42 (0.97)	NA	6.52 (0.91)	NA	-0.10 (-0.49 to 0.29)	NA	851.69	< 0.001	117.29	< 0.001	Baseline > 8 wk, 6 mo (*p* < 0.001 ^a,b^)	8 wk (*p* < 0.001); 6 mo (*p* < 0.001)	
8 weeks	2.52 (0.83)	60.74 ↓	4.52 (0.83)	30.67 ↓	-1.99 (-2.34 to -1.65)	2.41							
6 Month	3.58 (0.79)	44.23 ↓	5.61 (0.72)	13.95 ↓	-2.03 (-2.35 to -1.72)	2.69							
ODI (0-100)													
Baseline	39.80 (8.02)	NA	39.04 (8.40)	NA	0.76 (-2.67 to 4.18)	NA	414.15	< 0.001	85.84	< 0.001	Baseline > 8 wk, 6 mo (*p* < 0.001 ^a,b^)	8 wk (*p* < 0.001); 6 mo (*p* < 0.001)	
8 weeks	18.71 (4.49)	52.98 ↓	28.38 (4.09)	27.30 ↓	-9.67 (-11.46 to -7.88)	2.15							
6 Month	19.84 (2.29)	50.15 ↓	33.92 (2.35)	13.11 ↓	-14.08 (-15.05 to -13.11)	6.07							
TSK (17–68)													
Baseline	43.42 (5.94)	NA	44.50 (5.64)	NA	-1.08 (-3.49 to 1.34)	NA	539.37	< 0.001	64.93	< 0.001	Baseline > 8 wk, 6 mo (*p* < 0.001 ^a,b^)	8 wk (*p* < 0.001); 6 mo (*p* < 0.001)	
8 weeks	24.05 (3.78)	44.61 ↓	33.31 (3.71)	25.14 ↓	-9.27 (-10.83 to -7.70)	2.47							
6 Month	27.47 (3.84)	36.73 ↓	38.87 (2.89)	12.65 ↓	-11.39 (-12.81 to -9.98)	3.35							

**Abbreviations**: a: Results of Bonferroni Post Hoc tests in the CFT group; b: Results of Bonferroni Post Hoc tests in the MSI group; CI, Confidence Interval; NRS: numeric rating scale; NA, Not applicable; ODI: Oswestry Disability index; TSK: Tampa Scale of Kinesiophobia; ↓ decrease, ↑ increase; †, Effect size (Cohen’s *d*).

All calculations were done using intention-to-treat principles. Two-way repeated-measures analysis of variance (group × time) was used to compare differences between two groups based on adjusted analysis.

Table 3 showed that for VGRF parameters at the self-selected speed and faster speed, the FPF _self−selected speed_*(F = 65.51, p < 0.001)*, SPF _self−selected speed_*(F = 60.40, p < 0.001)*, MSF _self−selected speed_*(F =* 30.11, p < 0.001*)*, WAR _self−selected speed_*(F =* 22.06, p < 0.001*)*, POR _self−selected speed_*(F* 8.86, p < 0.001*)*, FPF _faster speed_*(*F *=* 27.91, p < 0.001*)*, WAR _faster speed_*(*F *=* 10.90, p < 0.001*)*, and POR _faster speed_*(F =* 125.57, *p <* 0.001*)* showed significant group × time interaction effects. Bonferroni post hoc analysis showed higher scores in the CFT group on the GRF parameters at self-selected speed and GRF parameters at faster speed at 8 weeks’ post-intervention, and 6 months follow-up. No statistically significant interaction between interventions was.

**Table 3:** Changes related to the scores of the GRF parameters

Characteristic	CFT		MSI		Group Difference, Mean (95% CI)	Effect Size^†^	Main Effect of Time		Interaction Effect		Bonferroni post-hoc tests	
	Means (± SD)	Change relative to baseline (%)	Means (± SD)	Change relative to baseline (%)			F	*P-*value	F	*P-*value	Time	Group
**GRF parameters at self-selected speed**												
FPF (%BW)												
Baseline	0.99 ± 0.03	NA	0.98 ± 0.03	NA	0.01 (-0.01 to 0.02)	NA	168.94	< 0.001	65.51	< 0.001	Baseline > 8 wk, 6 mo (*p* < 0.001^a,b^)	8 wk (*p* < 0.001); 6 mo (*p* < 0.001)
8 weeks	1.05 ± 0.02	6.06 ↑	0.99 ± 0.02	1.02 ↑	0.07 (0.05 to 0.07)	3						
6 Month	1.06 ± 0.02	7.07 ↑	0.99 ± 0.02	1.02 ↑	0.07 (0.06 to 0.08)	3.5						
SPF (%BW)												
Baseline	1.09 ± 0.02	NA	1.08 ± 0.03	NA	0.01 (0.00 to 0.02)	NA	30.53	< 0.001	60.40	< 0.001	Baseline > 8 wk (*p* < 0.001^a^), 6 mo (*p* < 0.001^a^) Baseline > 8 wk (N.S^b^), 6 mo (*p* = 0 .01^b^)	8 wk (*p* = 0.04); 6 mo (*p* < 0.001)
8 weeks	1.06 ± 0.02	2.75 ↓	1.07 ± 0.02	0.92 ↓	-0.01 (-0.02 to -0.005)	0.5						
6 Month	1.05 ± 0.02	3.67 ↓	1.09 ± 0.03	0.92 ↑	-0.04 (-0.06 to -0.03)	1.57						
MSF (%BW)												
Baseline	0.87 ± 0.02	NA	0.86 ± 0.02	NA	0.01 (0 to 0.02)	NA	165.18	< 0.001	30.11	< 0.001	Baseline > 8 wk (*p* < 0.001^a,b^), 6 mo (*p* < 0.001^a^), (*p* = 0.029^b^)	8 wk (*p* < 0.001); 6 mo (*p* < 0.001)
8 weeks	0.82 ± 0.03	5.75 ↓	0.84 ± 0.03	2.33 ↓	-0.02 (-0.03 to -0.01)	0.67						
6 Month	0.80 ± 0.02	8.05 ↓	0.83 ± 0.03	3.49 ↓	-0.03 (-0.04 to -0.02)	1.18						
WAR (%BW/s)												
Baseline	449.36 ± 37.48	NA	443.46 ± 45.39	NA	5.90 (-11.46 to 23.26)	NA	310.99	< 0.001	22.06	< 0.001	Baseline > 8 wk, 6 mo (*p* < 0.001 ^a,b^)	8 wk (*p* < 0.001); 6 mo (*p* < 0.001)
8 weeks	568.31 ± 31.87	26.47 ↑	511.66 ± 41.30	15.38 ↑	56.65 (41.26 to 72.04)	1.53						
6 Month	563.66 ± 26.33	25.29 ↑	510.51 ± 36.70	15.12 ↑	53.15 (39.82 to 66.48)	1.66						
POR (%BW/s)												
Baseline	619.49 ± 48.12	NA	615.27 ± 53.72	NA	3.77 (-17.50 to 25.04)	NA	220.59	< 0.001	8.86	< 0.001	Baseline > 8 wk, 6 mo (*p* < 0.001 ^a,b^)	8 wk (*p* < 0.001); 6 mo (*p* = 0.001)
8 weeks	750.14 ± 58.11	21.09 ↑	702.75 ± 59.95	14.22 ↑	47.39 (22.79 to 71.99)	0.80						
6 Month	749.79 ± 61.77	21.03 ↑	702.49 ± 69.69	14.18 ↑	47.30 (19.85 to 74.76)	0.72						
**GRF parameters at faster speed**												
FPF (%BW)												
Baseline	1.09 ± 0.03	NA	1.08 ± 0.03	NA	0.01 (-0.01 to 0.02)	NA	107.41	< 0.001	27.91	< 0.001	Baseline > 8 wk, 6 mo (*p* < 0.001 ^a,b^)	8 wk (*p* < 0.001); 6 mo (*p* < 0.001)
8 weeks	1.15 ± 0.03	5.50 ↑	1.11 ± 0.03	2.77 ↑	0.04 (0.02 to 0.05)	1.33						
6 Month	1.15 ± 0.02	5.50 ↑	1.09 ± 0.02	0.93 ↑	0.06 (0.05 to 0.07)	3.00						
SPF (%BW)												
Baseline	1.14 ± 0.03	NA	1.13 ± 0.03	NA	0.01 (-0.004 to 0.023)	NA	21.53	< 0.001	0.39	0.68	Baseline > 8 wk (*p* < 0.001^a,b^)	8 wk (N.S); 6 mo (*p* = 0.032)
8 weeks	1.16 ± 0.03	1.75 ↑	1.16 ± 0.03	2.65 ↑	0.004 (-0.01 to 0.02)	0						
6 Month	1.15 ± 0.02	0.88 ↑	1.14 ± 0.02	0.88 ↑	0.01 (0.001 to 0.02)	0.5						
MSF (%BW)												
Baseline	0.65 ± 0.03	NA	0.67 ± 0.03	NA	-0.01 (-0.03 to 0)	NA	8.53	< 0.001	0.86	0.42	Baseline > 6 mo (*p* < 0.001^a^)	8 wk (*p* = 0.01); 6 mo (*p* < 0.001)
8 weeks	0.64 ± 0.03	1.54 ↓	0.66 ± 0.04	1.49 ↓	-0.02 (-0.03 to -0.005)	0.57						
6 Month	0.63 ± 0.03	3.07 ↓	0.66 ± 0.03	1.49 ↓	-0.02 (-0.04 to -0.01)	1						
WAR (%BW/s)												
Baseline	1157.49 ± 124.11	NA	1134.22 ± 117.22	NA	23.27 (-27 to 73.55)	NA	6.17	< 0.003	10.90	< 0.001	Baseline < 8 wk(*p* < 0.001^a^), 6 mo (*p* = 0.048^a^) Baseline < 8 wk(N.S^b^), 6 mo (*p* = 0.009^b^)	8 wk (*p* = 0.02); 6 mo (*p* < 0.001)
8 weeks	1235.64 ± 111.23	6.75 ↑	1156.86 ± 124.06	2.00 ↑	78.78 (29.66 to 127.90)	0.67						
6 Month	1232.08 ± 120.21	6.05 ↑	1041.90 ± 251.77	8.13 ↓	190.18 (107.7 to 272.66)	0.96						
POR (%BW/s)												
Baseline	992.53 ± 50.34	NA	989.37 ± 45.12	NA	3.16 (-16.74 to 23.07)	NA	290.78	< 0.001	125.57	< 0.001	Baseline < 8 wk, 6 mo (*p* < 0.001^a,b^)	8 wk (*p* < 0.001); 6 mo (*p* < 0.001)
8 weeks	1156.24 ± 43.94	16.49 ↑	1038.86 ± 36.37	5 ↑	117.37 (100.59 to 134.16)	2.91						
6 Month	1155.87 ± 38.77	16.46 ↑	1009.18 ± 48.38	2 ↑	146.69 (128.40 to 164.98)	3.35						

**Abbreviations**: a: Results of Bonferroni Post Hoc tests in the CFT group; b: Results of Bonferroni Post Hoc tests in the MSI group; CI, Confidence Interval NA, Not applicable; N.S, Not significant; NRS: numeric rating scale; ODI: Oswestry Disability index; TSK: Tampa Scale of Kinesiophobia; ↓ decrease, ↑ increase; †, Effect size (Cohen’s *d*).

All calculations were done using intention-to-treat principles. Two-way repeated-measures analysis of variance (group × time) was used to compare differences between two groups based on adjusted analysis.

### Adverse events

The CFT and MSI treatment groups did not report any adverse events during the eight-week training, and the assessments in the pre-test, post-test, and 6-month follow-up did not record any adverse events.

## Discussion

This study aimed to compare efficacy of CFT and MSI-based treatment on pain, disability, kinesiophobia, and gait kinetics in CNSLBP patients. Evaluation of functional status revealed superiority of CFT approach on reduction in pain intensity, disability, and kinesiophobia through an 8-week intervention and six months follow up over MSI-based treatment.

According to the bio-psycho-physiological improvement in recent years, it is time for health-care practitioners to shift their perspective away from a biomedical model, toward prescribing the treatment for CNSLBP based on a multidimensional classification-based approach with the aim of making the treatment more self-centered and focusing on cognitive-behavioral aspects rather than the signs and symptoms associated with the disorder.

According to O’Sullivan’s classification, two sensitizations found LBP, including central sensitization affected by psychological and cognitive factors, and peripheral sensitization connected to impairments in movement and controlling movement [33]. CFT in its own way and also in our study emphasized on both psychological and movement-related factors and covered both sensitizations. It achieved by providing a new understanding of the patients’ condition and increase patients’ understanding about their old beliefs, teaching patients how to improve their daily movements, and changing their lifestyle toward a healthier one with more self-confidence and self-efficacy. However, MSI-based treatment mostly covered the diagnosed movement impairments and teaches patients how to control their movement; while, does not fully target the psychological and cognitive factors. Therefore, although this is not clear as to the exact basis for the superior outcomes because CFT is multidimensional approach, it can be hypothesized that the superiority of CFT on MSI-based treatment on pain intensity, disability, and kinesiophobia can be associated with the fact that CFT can support both psychological- and movement-based aspects of CNSLBP.

Activity avoidance is related to fear, which reflects the belief that activity may result in (re)injury or increased pain as a result of dysfunctional interpretations of pain and injury that can be adaptive in the acute pain stage but contradictorily worsen the problem in the case of chronic pain [35]. The use of a qualitative element via clinical interviews showed that being educated reduces pain and fear of movement, providing insight into patients’ perspective on this process [36]. CFT may speculate to lead an individual’s perspective to a rapid disruption and guide their behavior toward positive beliefs, enhanced understanding, and control of pain, improved self-efficacy, confidence, and mood. Evidence supported the reduction in fear of movement and observed improved mood following the CFT approach [37].

To support our results, Vibe Fersum et al. (2013) [38] stated that CFT can produce a statistical and clinical superiority on combined manual therapy and exercise for reducing pain intensity (with 3.2 points of improvement compared to 1.5), disability (with 13.7 points of improvement compared to 5.5), and fear avoidance belief in patients with CNSLBP with 3- to 12-month follow-up maintenance. Caneiro et al. (2017) [36] in their single case report, showed improvement in pain expectancy, pain experience, and kinesiophobia in a male subject after 6 sessions in a 3-month period of CFT.

After 8 weeks of treatment with both interventions, the evaluation of gait kinetics in people with CNSLBP showed significant improvements in VGRF parameters such as FPF, SPF, MSF, WAR, and POR from baseline. However, the CFT approach appeared to be more effective than the MSI-based treatment. The results showed that, the POR and WAR increased in the self-selected speed in both treatment groups. It means that the time to reach heel-contact and return time from push off have decreased [39]. Thus, it may show that patients walked faster after both intervention; however, it was faster in CFT group. The difference between the two peaks was greater, the SPF was higher than the FPF, and both VGRF peaks were shorter at baseline for both groups. At post-treatment, the difference between FPF increased and SPF decreased more for the CFT group than for the MSI-based treatment group. In other words, the SPF peaks got closer to the FPF peaks after the 8-week CFT intervention. MSF was higher in midstance at the baseline but this number decreased after the 8-week interventions, especially in the CFT group. It seems that CNSLBP patients used their muscles more efficiently in midstance after the interventions.

At the self-selected and fastest walking speeds, WAR and POR increased in the CFT group more than in the MSI group, representing increased speed and decreased time in reaching the FPF peak and returning from the SPF peak. FPF and SPF both increased at the fastest walking speeds in both intervention groups. Generally, more improvement in MSF was observed in the CFT group. To the best of our knowledge, this is the first trial assessing the effects of CFT and MSI-based treatment on gait kinetics in patients with CNSLBP. Our results clearly showed that impaired gait in CNSLBP patients improved more in the CFT group than in the MSI group.

Previously, Barzilay et al. (2016) [40] found that a home-based biomechanical treatment combining the use of foot-related biomechanical device into the patient’s daily routine can improve gait spatiotemporal parameters in CNSLBP patients. The authors also found that the improvement of disability using ODI was associated with improvement of gait parameters. These authors suggested a combined intervention targeting both physical and behavioral aspects.

To support Barzilay’s study findings, we found the improvement of VGRF parameters with the improvement of pain, disability, and kinesiophobia. In CFT group we saw significant percentage of changes for pain (60.74%), disability (52.98%), and kinesiophonbia (44.6%) accompanied with the better improvement of VGRF parameters after prescribing eight-week multidimensional intervention. Also, significant percentage of changes for pain (44.23%), disability (50.15%), kinesiophonbia (36.73%) and improvement of VGRF parameters observed in CFT group six months after the first treatment.

According to Krekoukias et.al [41], LBP patients are able to walk faster than their preferred velocity, but they prefer to walk with a lower velocity, the reason is related to inability to dealing with perturbations. In theory, altering muscle activity can regain spinal stability, but these changes may cause microtrauma, alter sensory input, increase spinal instability, and decrease the ability to deal with perturbations, which results in diminished anticipatory behavior in the case of balance loss. Therefore, patients with LBP try to walk slowly to have more control on their movements [41]. On the other hand, Lee et al. found that low back pain patients reduced the push-off rate compared to healthy persons [4]. In another study, researchers showed that patients choose to walk at a slower speed regardless of the distribution of pain, which they suggested decreases the ground reaction force [42].

In this study patients in both experimental groups preferred to walk faster in post-test in both self-selected speed and faster speed, besides the push-off rate improved in both groups specially in CFT group. Therefore, it is likely cognitive functional therapy improved the dealing with perturbations and as a result progressed the push-off rate and gait speed.

Patients with low back pain, showing higher electromyographic activity of the rectus abdominis and erector spinae during gait [43]. Also, by increasing walking speed, pelvis-trunk complexes of low back pain patients showed reduced adaptability [44]. in this study it’s possible that, CFT group had better adaptability in the pelvis-trunk complexes during faster walking after eight-week training and after 6 month follow up.

In contrast to the physiological gait, the pelvis rotates in the opposite direction from the trunk, but low back pain patients demonstrated gait compensations that exhibit increased pelvic rotation with concurrent trunk rotation which is assumed for antalgic purposes [45].

In this study, the CFT group showed better pain improvement compared to the MSI group, which may result in a greater reduction of these compensatory movements. The CFT group has demonstrated more progression in gait parameters.

Other aspects include the influence doctors have on patient outcomes and treatment confirmation, including their communication skills, empathy and degree of trust [2]. Along with patient participation, knowledge of activity is crucial for reducing patients pain and normalizing a their activity, The how patients engaged in cognitive functional treatment to desensitize central sensitization changes determines how effective it is for postural control. With continuing peripheral nociceptor input from the intervertebral disc, pain produced in the forebrain is less amplified to the central nervous system [2].

similar to our previous hypothesis, we can draw a conclusion that when patients received both psychological- and movement-based treatment that educated patients how to move better in their daily-routines, overcome their fear and change their beliefs toward a self-control management, they can perform better their functional tasks like walking with less fear of movement.

This study has some limitations First, to better evaluate gait performance, researchers propose including not only VGRF but also the horizontal ground reaction forces. Second, the current study is limited by the lack of electromyography (EMG) in recording the functioning of core and abdominal muscles. Third, future studies should examine CFT intervention with other therapeutic methods, as it is currently compared with MSI treatment in this study. Fourth, this study based its sampling strategy on patients with chronic low back pain, which is defined as pain lasting for 90 days or more. Although the researchers selected pain intensity as the primary outcome measure, it should be noted that experts often recommend studies of patients with chronic pain to prioritize the primary outcome of disability, as pain intensity is more susceptible to regression to the mean. Despite using validated measurement tools and baseline assessments to minimize this potential limitation, the decision to make pain intensity the primary outcome may influence the overall interpretation of treatment efficacy. Finally, the lack of a translated and validated Persian version of Central Sensitization Inventory prevented us from assessing central sensitization. If we had used this tool, we could have determined whether there was any association between pain improvement and central sensitization in the studied population. For future similar studies, a translated and validated version of the Persian Central Sensitization Inventory will be beneficial, in addition to other pain intensity measures. For future studies, authors also suggest that random forest analysis with the Shapley Additive Explanations (SHAP) summary plot can present useful information regarding the rankings of major predictors and the directions of their associations with the superior effects of cognitive functional therapy.

## Conclusions

The main implication of the current findings is that, in addition to changing lifestyle and functional training, fostering a biopsychosocial understanding of pain and cognitive training are helpful for maximizing efficiency of treatment in patients with non-specific chronic LBP. The current data suggest that both CFT and MSI-based treatment seem to have beneficial effects on rehabilitation of CNSLBP patients through reduction of pain, disability, and fear-avoidance behaviors, and improvement of VGRF parameters. The CFT approach produces better outcomes, which may be due to its multidimensional therapeutic effect. Of course, the results of this study need to be confirmed with a larger number of patients.

### Electronic supplementary material

Below is the link to the electronic supplementary material.

Supplementary Material 1

Supplementary Material 2

## Data Availability

The datasets used and analysed during the current study available from the corresponding author on reasonable request.

## List of abbreviations

CFT: cognitive functional therapy
MSI: movement system impairment
CNSLBP: non-specific low back pain
LBP: Low-back pain
NRS: Numeric Ratings Scale
ODI: Oswestry disability index
TKI: Tampa Kinesiophobia Scale
RCT: randomized controlled trial
[ICD-10]: International Classification of Diseases, 10th Revision
VGRF: vertical ground reaction force
FPF: First Peak Force
SPF: second Peak Force
MSF: Mid-Support Force
WAR: Weight Acceptance Rate
POR: Push-Off Rate
